# A graphene-coated AFM probe for durable and reproducible nanoscale electronic measurements

**DOI:** 10.1039/d5na00924c

**Published:** 2026-03-04

**Authors:** Xintai Wang, Angelo Lamantia, Becky Penhale-Jones, Nema Abdelazim, Oleg. V. Kolosov, Benjamin. J. Robinson

**Affiliations:** a Physics Department, Lancaster University Lancaster LA1 4YB UK b.j.robinson@lancaster.ac.uk; b Zhejiang MaShang GM2D Research Institute Cangnan Wenzhou Zhejiang China; c School of Information Science and Technology, Dalian Maritime University Dalian China; d School of Electronics and Computer Science, University of Southampton Southampton SO17 1BJ UK

## Abstract

Conductive atomic force microscopy (cAFM) is a powerful tool for investigating electronic and thermoelectric properties at the nanoscale. However, the widespread application of cAFM is hindered by the rapid wear and unpredictable failure of metal-coated probes, leading to poor measurement reproducibility and limited probe lifetime. Here, we report a scalable fabrication method for graphene-coated cAFM probes using the Langmuir–Blodgett technique. These probes exhibit exceptional mechanical durability, including resistance to both friction-induced wear and high-current stressing, and maintain stable electrical performance over extended use. When applied to self-assembled monolayers (SAMs), the graphene-coated probes yield narrow conductance distributions, significantly improved measurement reproducibility across different probe batches, and a substantial reduction in short-circuit artifacts. The graphene coating also provides a more compliant tip-sample contact, minimizing damage to soft molecular layers. Electronic transport and thermoelectric measurements further confirm the reliability of these probes, revealing tunnelling characteristics and Seebeck coefficients consistent with established values. Our work establishes a robust and scalable platform for nanoscale electrical characterisation, overcoming a critical limitation in conventional cAFM and opening avenues for long-term, reproducible studies in molecular electronics and beyond.

## Abstract 2

Graphene-coated cAFM probes fabricated *via* Langmuir–Blodgett assembly exhibit superior mechanical durability and stable electrical performance, enabling reproducible nanoscale transport and thermoelectric measurements on molecular systems.

## Graphical abstract

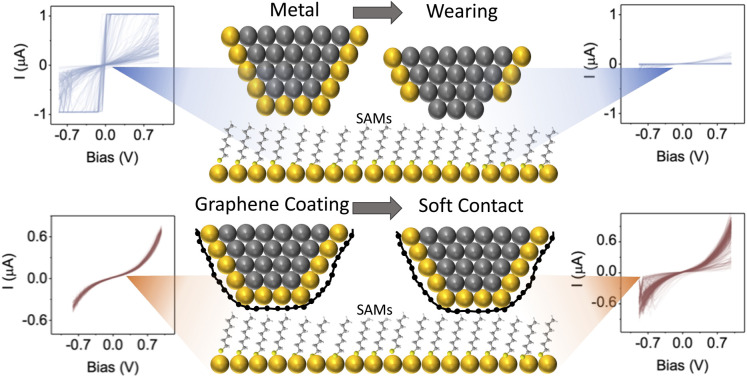

## Introduction

Molecular electronics, which utilises molecules as the fundamental building blocks for electronic devices, has been an active area of research for over 40 years.^1–8^ In the past two decades, extensive theoretical and experimental studies have been conducted in this field.^2,3,9–14^ Various techniques, including scanning tunnelling microscopy (STM)^15,16^ and mechanically controllable break junctions (MCBJs),^17–19^ have been employed to study single-molecule junctions. Additionally, large-scale molecular junctions have been investigated using Eutectic Gallium–Indium (EGaIn) junctions and microchip devices.^5,20–23^ Among these techniques, conductive atomic force microscopy (cAFM) has emerged as a powerful approach for studying quantum transport and thermoelectric properties in molecular junctions.^4,6,24–29^

Most cAFM measurements utilise noble metals like platinum or gold to coat the probe to create conductive electrode. However, wear of these metal coatings due to shear force in the junction remains a challenge, leading to poor and changing contact, significant measurement variation, and open circuits.^30,31^ While some wear-resistant probes, such as highly doped diamond^32,33^ or PtSi-coated probes,^34–36^ exist, their thick coating layers degrade AFM resolution and increase costs. Graphene, an ultra-thin and highly conductive material, presents a promising solution to these challenges when used as a coating layer for cAFM probes.^30,31,37^ Additionally, the graphene film is expected to reduce variability in the probe-sample contact area arising from nanoscopic roughness and wear at the metallic probe apex.^30^ Previous graphene-coating techniques, such as chemical vapor deposition (CVD) growth,^30^ polymer-assisted graphene transfer,^37^ and dip coating,^31^ face issues like low coating yield, deposition control challenges, polymer residues, and fabrication complexity.

In this work, we introduce a straightforward method for transferring ultra-thin graphene films to the apex of cAFM probes using the Langmuir–Blodgett (LB) film forming technique. The LB approach uses the surface tension of a water subphase to align, typically, amphiphilic or hydrophobic materials in a highly ordered monolayer at the air–water interface and subsequently transfer this monolayer *via* perpendicular (also known as LB transfer) or parallel (Langmuir–Schaffer (LS)) transfer to a chosen solid substrate. Controlled multilayer formation can be achieved through repeated transfer steps.

**Fig. 1:**
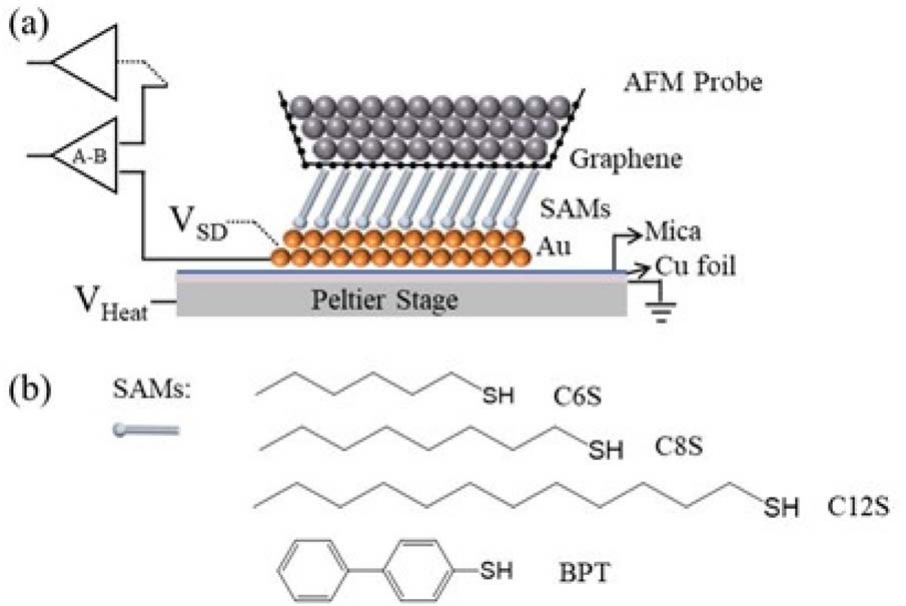
Schematic of the graphene coated conductive AFM experimental setup (a) and measured molecules (b).

This method enables the scalable production of uniformly coated probes with high reproducibility, addressing the limitations of existing approaches. We then employ graphene-coated probes to investigate the electrical and thermoelectric properties of self-assembled monolayer (SAM) systems (Fig. 1), demonstrating their improved performance over probes without a graphene coating.

## Experimental section

### Graphene film preparation and transfer

Here, we will use the convention of the terms “deposit” and “deposition” to refer to the spreading and formation of the graphene film on the water subphase, and the term “transfer” for the subsequent coating of the deposited film from the air–water interface to the substrate or probe (illustration as Fig. S1(a)). A dispersion of graphene flakes (Sigma-Aldrich, 0.2 mg mL^−1^, 1–3 layers) in dimethylformamide (DMF) was evenly deposited onto a 750 cm^2^ Langmuir–Blodgett (LB) trough (KSV NIMA) filled with ultrapure DI water as the subphase. The dispersion was applied *via* syringe, with each 15 µL droplet spaced by a 1 minute interval. The water's surface tension (72 mN m^−1^) maintained the graphene flakes as discrete islands on the surface. A Wilhelmy plate, butane flame-cleaned, was used to measure surface tension.

The KSV NIMA control system regulated the movement of two barriers, compressing the graphene islands at a constant rate of 5 mm min^−1^. The surface pressure–area (π–A) isotherm was recorded in real-time, while Brewster angle microscopy (BAM) was used to monitor surface morphology at varying surface pressures.

Substrates, including cleaned Si wafers and conductive AFM Si probes (Multi-75E, Pt/Cr coated, Budget Sensors) mounted on Si wafers using Gel-Pac™ glue (adhesive strength X0), were thoroughly cleaned *via* sequential rinses in methanol, ethanol, and isopropanol, followed by air drying and oxygen plasma treatment (800 W, 30 s).

Graphene LB films formed at various surface pressures were transferred onto these prepared substrates using the Langmuir–Schaefer (LS) method.^38,39^ Transfer parameters were set at a dipping speed of 2 mm min^−1^ and a lifting speed of 10 mm min^−1^. After transfer, samples were dried in a vacuum oven at 80 °C for 2 hours.

### Self-assembled monolayer (SAM) formation

SAMs were assembled on template-stripped gold (Au^TS^) surfaces. Si wafers (5 mm × 5 mm) were cleaned by sequential ultrasonication in acetone, methanol, and isopropanol, followed by oxygen plasma cleaning for 5 minutes. Cleaned wafers were bonded to a thermally evaporated gold layer (100 nm thick on a separate Si substrate) using Epotek 353ND epoxy adhesive, forming a Si/epoxy/Au/Si sandwich structure. The adhesive was cured at 150 °C for 40 minutes. After curing, the bottom Si layer was removed using a razor blade to expose an atomically flat Au surface. AFM imaging was performed on 3–5 random regions of the prepared gold surface for quality assessment. Only substrates with root-mean-square roughness below 70 pm were used for subsequent self-assembled monolayer (SAM) formation.

Self-assembled monolayers (SAMs) were formed from 100 µM solutions of target molecules dissolved in ethanol (alkyl thiols) or toluene (BPT). Solutions were deoxygenated by nitrogen bubbling for 10 minutes prior to use. All chemicals (Sigma-Aldrich™) were used as received. Freshly cleaved Au^TS^ substrates were immersed in the SAM solutions and incubated under nitrogen for 24 h. Post-incubation, alkyl thiol samples were rinsed with ethanol, while BPT samples underwent sequential rinsing with toluene and ethanol. All samples were nitrogen-dried and left in a vacuum oven (10^−2^ mbar) at 35 °C overnight for solvent removal.

### Atomic force microscopy methods

Graphene film morphology was characterised using a Bruker MultiMode with Nanoscope 8 controller atomic force microscope (AFM) in PeakForce tapping mode (force setpoint: 500 pN). Nano-scratching experiments were performed using a soft AFM probe (spring constant *k* = 0.3 N m^−1^) in contact mode under high normal force (15–40 nN), scanning a 300 nm × 300 nm area for several cycles. Post-scratch topography was imaged again in PeakForce mode. Bare gold substrates were subjected to identical scratching procedures to confirm that the applied forces did not damage the gold. The thickness of the SAM was determined from the height difference between scratched and unscratched regions.

Electrical characterisation of SAMs was conducted using a cAFM system based on the same MultiMode platform in contact mode. The bottom Au substrate acted as the source, and graphene-coated or uncoated cAFM probes served as the drain. A Keithley 2400 source meter applied voltage bias between the probe and sample, while a precision low-noise current I–V converter (FEMTO) was used to amplify and record current at the tip apex. Voltage bias was applied either as constant or swept values depending on experimental requirements.

The Seebeck coefficient of SAMs was measured using a thermal-electrical AFM (ThEFM) setup,^40^ modified from the cAFM system. Samples were placed on a Peltier stage with top electrically grounded through a metallic plate and thermally regulated using a Keithley 2400 source meter. A thermocouple provided real-time temperature monitoring. The AFM probes (graphene-coated and uncoated) contacted the sample surface under a constant force of 1.4 nN. Thermally induced voltages generated by the probe-sample temperature gradient were amplified using a Stanford Research Systems SR550 voltage preamplifier.

## Result and discussion

### Film characterisation

The graphene Langmuir–Blodgett (LB) film was fabricated by depositing a graphene dispersion in DMF solvent onto an ultrapure water subphase (see Experimental Section). The surface pressure–area isotherm is provided in the SI (Fig. S1b). Fig. S1c–h show Brewster angle microscopy (BAM) images acquired at progressively increasing surface pressures using a KSV NIMA MicroBAM instrument. This image series illustrates the structural transition from isolated graphene flake islands (Fig. S1c) to a continuous, densely packed thin film on the trough surface during barrier compression (Fig. S1g and h).

**Fig. 2:**
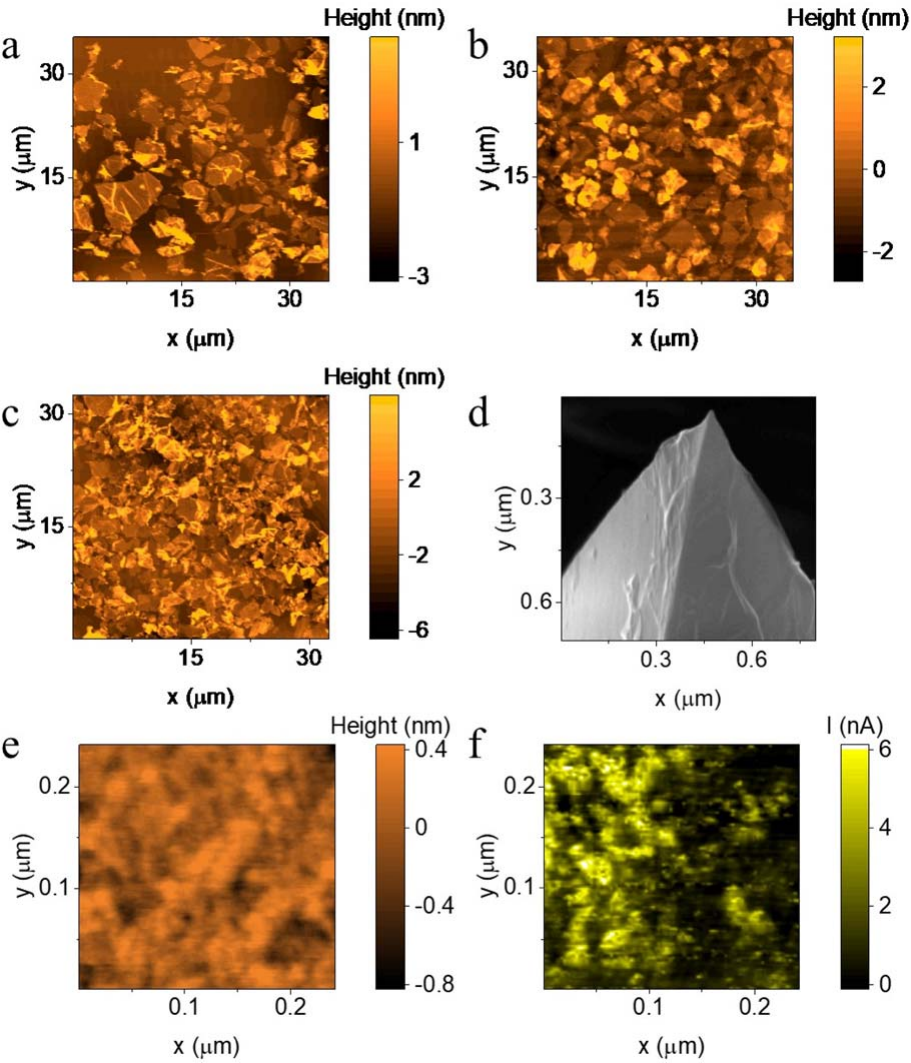
(a–c) AFM image of graphene LB film transferred onto a Si substrate while surface pressure is 6 N m^−1^, 8 N m^−1^ and 10 N m^−1^ (d) SEM image of graphene LB film transferred onto an AFM probe. (e and f) Topography and current image of biphenyl thiol (BPT) SAM on Au substrate scanned by graphene coated probe, scan size: 250 nm.

For initial assessment of film packing and morphology, the graphene film was transferred onto a planar Si substrate-by LS transfer at surface pressure of 6, 8 and 10 N m^−1^ and characterised by atomic force microscopy (AFM) imaging (Fig. 2(a–c)). A surface pressure of 8 N m^−1^ (Fig. 2(b)) gave the optimum balance of surface coverage (over ∼90% coverage, compared to ∼70% coverage at 6 N m^−1^), while maintaining a thin and uniform film thickness (film thickness ∼1.5–3 nm, illustrated in Fig. S2) as a thick graphene coating is undesirable for cAFM probes as it not only increases the probe's radius curvature, degrading AFM resolution, but also increases the number of graphene stacking interfaces, reducing probe's electric sensitivity.

### Probe characterisation

We compare Pt coated cAFM probes with and without a transferred graphene layer. Transfer of graphene films to the Pt coated cAFM probes was performed using the same deposition and 8 N m^−1^ surface-pressure transfer conditions detailed above. A total of 20 probes were coated in three batches, each batch used a fresh LB graphene film to test reproducibility between film deposition, demonstrating scalability for large-scale production. Scanning electron microscopy (SEM) images confirmed uniform graphene coverage without significant changes in probe apex radius (Fig. S3(a–d)).

Fig. 2(e) and (f) demonstrate the resolution test of the graphene-coated probe. From the results, it can be observed that without any additional noise-cancelling techniques, the probe after graphene coating exhibited good topographical resolution with a scan size less than 300 nm (sample: octanethiol (C8S)-based SAM on template-stripped Au substrate (Au^TS^)). This resolution is comparable to that achieved with a non-coated probe under the same scanning conditions, indicating that the graphene coating on AFM probes has minimal impact on lateral resolution. Notably, the SEM image of the graphene-coated probe (Fig. 2(d)) reveals distinct wrinkle structures. However, the current maps acquired on the AuTS surface (Fig. S11(a–d)), together with the corresponding statistical analysis (Fig. S11(e)), demonstrate that the measurement uniformity of the Pt probe remains largely unchanged with or without graphene coating. This indicates that morphological features of the graphene coating—including step edges and wrinkles—exert negligible influence on the measurement outcome. This can be attributed to the fact that these wrinkles originate primarily from the water-assisted transfer process and manifest mainly at the microscale. In contrast, nearly no nano-wrinkles are observed on the graphene flake itself, as evidenced in Fig. 2(a–c). Consequently, it is unlikely that such micro-wrinkles would cover the nanoscale apex of the probe during measurement.

### Wear resistance tests

The wearing of the AFM probe's metal coating is a significant challenge for cAFM, particularly during lateral scanning where the friction between the sample and probe can easily wear out the metallic coating, or in the presence of high current density^41^ arising from nanometre range junction, leading to the burnout of the metal coating and significant decrease the probe's lifespan.

**Fig. 3:**
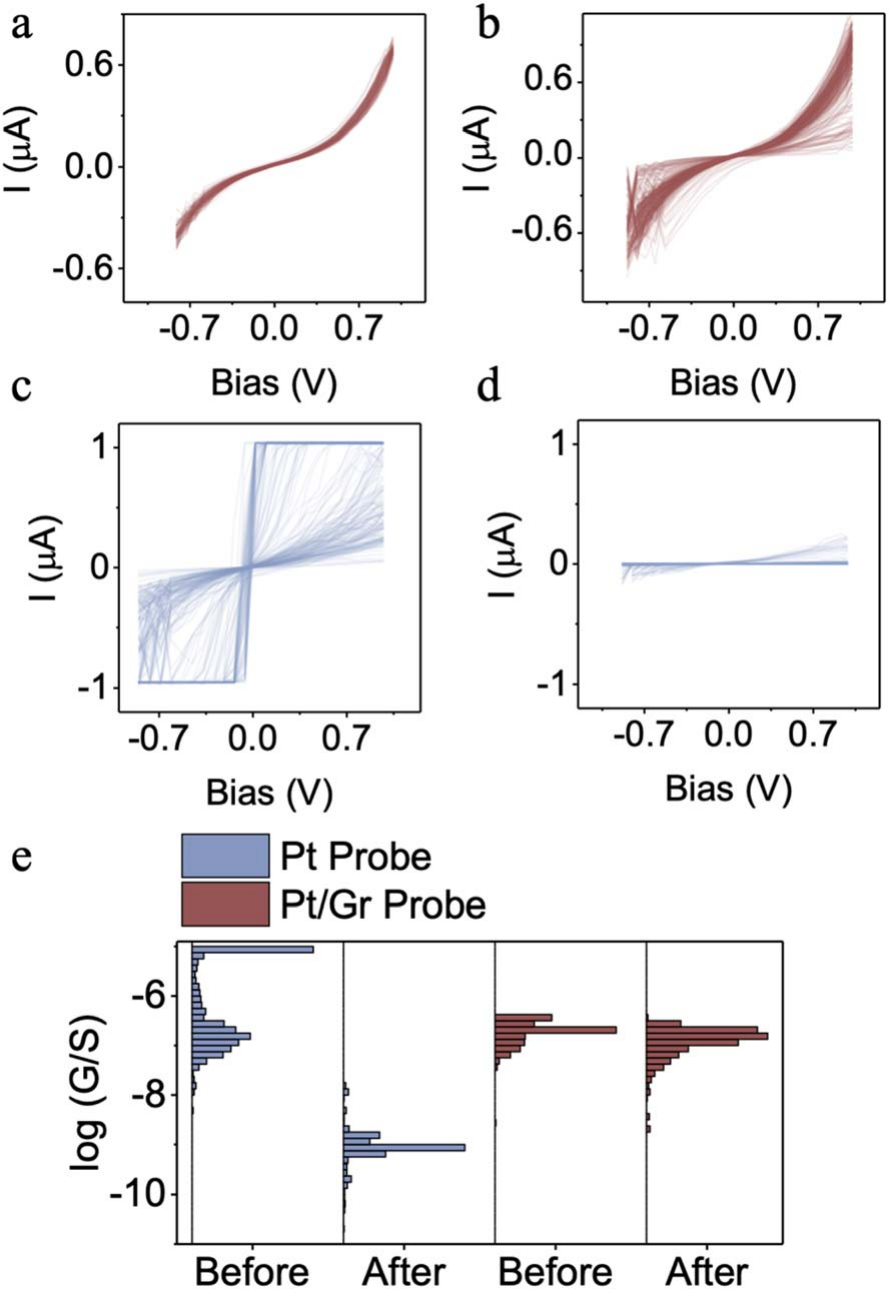
(a and c) IV curve of BPT SAMs measured by cAFM using a new cAFM probe with and without graphene coating. (b and d) Same as (a and c), but the cAFM probe was after 5 image scan cycles (lateral size: 500 nm, force: 2 nN). (e) The distribution of conductance before and after image scan measured by probe with and without graphene coating.

To evaluate friction-induced wear resistance, a newly prepared graphene-coated probe was employed to measure the I–V characteristics of a biphenyl thiol (BPT) SAM on a gold substrate. After multiple scanning cycles, the IV curve (Fig. 3(a)) and conductance distribution remained unchanged (Fig. 3(e)), indicating no wearing of the graphene-coated probe. Subsequently, the probe underwent five image scans with a lateral size of 500 nm in contact mode, before the electrical characterisation was repeated^42^ (Fig. 3(b) and (e)), again no significant change in the probe's electrical sensitivity was observed. In contrast, Pt-coated probes exhibited a significant decrease in current intensity under the same test condition (Fig. 3(c–e), left) before and after the image scan, suggesting that wearing of the conductive layer had occurred.

Post-scanning SEM characterisation of both coated and uncoated probes further supports this conclusion. Pt-coated probes exhibit distinct wearing phenomena (Fig. S4, denoted by black arrows), which are absent in graphene-coated counterparts. Furthermore, conductive AFM current mapping demonstrates pronounced signal attenuation in uncoated probes, whereas graphene-modified probes show negligible current decay (Fig. S6).

As shown in Fig. 3(c), a number of short-circuit IV curves are observed when using probes without a graphene coating. These are characterised by the current reaching the detection limit at very low bias voltages, indicating the formation of a metal–metal junction. This occurs because the rigid probe penetrates the delicate SAMs layer. In contrast, when the probes are coated with graphene, the film serves as a compliant and low friction interlayer between the probe and the SAMs, effectively minimising probe penetration. Consequently, no short-circuit IV curves appear in Fig. 3(a). The compliant nature of the graphene-mediated contact is further corroborated by nanomechanical mapping. Template-stripped Au substrates were scanned in PeakForce Quantitative Nanomechanical Mapping (QNM) mode using both Pt-coated and graphene-coated Pt probes. Over a 1 µm × 1 µm area (256 × 256 pixels), force–distance curves were acquired at a force setpoint of 0.5 nN at each pixel, and Young's modulus values were extracted *via* DMT fitting of the repulsive region spontaneously. The distribution of moduli (Fig. S5) shows a clear reduction from an average of ∼6.3 × 10^10^ Pa for the bare Pt probe to ∼5 × 10^9^ Pa for the graphene-coated probe, confirming that the graphene layer softens the mechanical interaction and helps prevent penetration of the molecular monolayer.

**Fig. 4:**
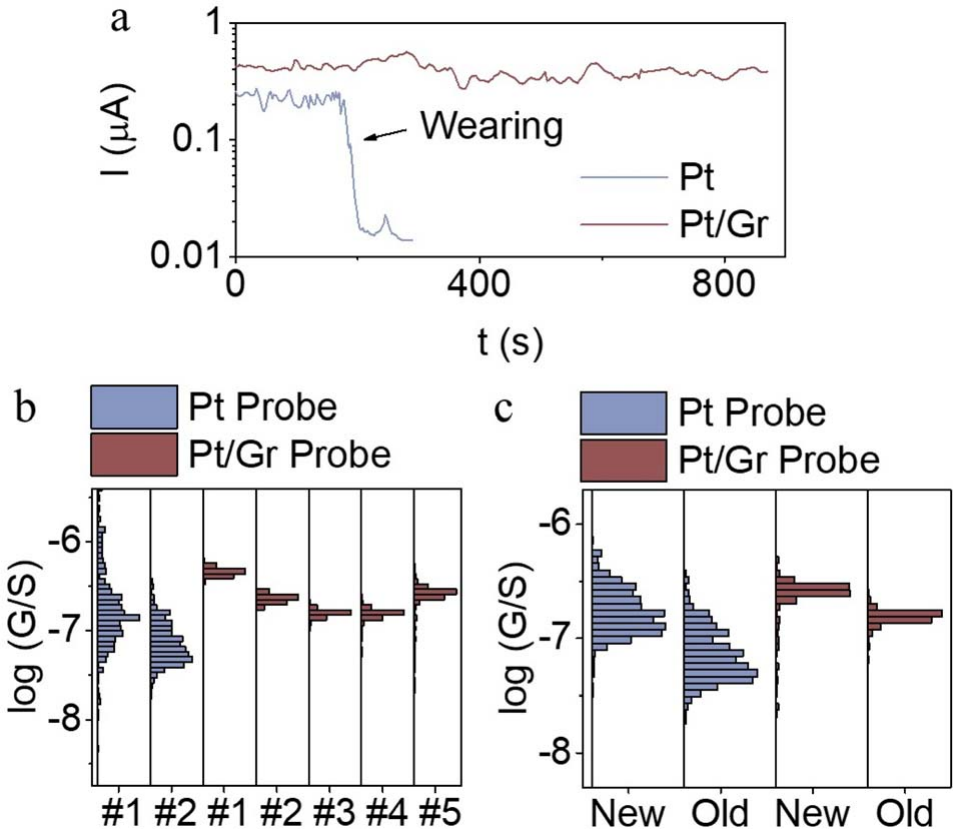
(a) Lifetime measurement of Pt probes with and without graphene coating, measured target: BPT SAM on Au^TS^, bias voltage = 0.7 V. (b) Measured conductance distribution of BPT SAM with fresh prepared probe (denote as new) and the same probe leave in air for 1 month (denote as old) with and without graphene coating. (c) Measured conductance distribution of BPT SAM with Pt probe and graphene coated probe prepared from different batch.

For high-current-induced wear tests, a constant bias of 0.7 V was applied to probes in contact with the BPT SAM at a constant set force of 2 nN (Fig. 4(a) and S10). A total of 8 probes were tested, including 4 with graphene coating and 4 without graphene coating (Fig. 4 and S10). All graphene-coated probes exhibited stable current responses for at least 15 minutes. In contrast, uncoated probes showed a significant current decrease within 2–4 minutes, with the current dropping by approximately an order of magnitude (*I*/*I*_initial_ ∼0.1) (Table S1). These results demonstrate the superior durability of the graphene-coated probes.

### Reproducibility and stability

To assess the reproducibility of measurements across different probes, the same BPT sample was tested using graphene-coated probes from various preparation batches, all fabricated using the same procedure. As shown in Fig. 4(b), conductance distributions measured by five different graphene-coated probes (labelled #1–#5 in cyan) exhibited narrow variation, with average conductance values falling within the same order of magnitude and a full width at half maximum (FWHM) below 0.2. This indicates a high degree of measurement reproducibility with probes prepared using this method. For comparison, two freshly prepared probes without graphene coatings (labelled #1 and #2 in red) were used to measure the same sample. Although their average conductance was within the same order of magnitude, the distribution was significantly broader (FWHM > 0.5), likely due to variability at the contact area at the metal–organic interface due to uncertain probe apex structure^30,43^ which is eliminated by the introduction of the uniform graphene film.

Fig. 4(c) further demonstrates the long-term stability of graphene-coated probes. Conductance measured using a newly prepared graphene-coated probe (“new”) closely matched that of the same probe after one month of air exposure (“old”), with nearly identical results (Fig. 4(c), cyan). However, a bare Pt probe exhibited a threefold reduction in measured conductance after similar air exposure (Fig. 4(c), red). This degradation is likely due to the hydrophilic nature of Pt, which promotes the formation of a thin water layer that can trap airborne contaminants, leading to performance deterioration. In contrast, the hydrophobic nature of graphene^44,45^ prevents such water layer formation, thereby maintaining probe cleanliness and performance. Supporting this, Fig. S6 shows current maps of the BPT sample at ±500 mV using a probe stored in air for one month. The graphene-coated probe produced uniform current across the scan area, while the uncoated probe displayed numerous discontinuous regions, indicative of contamination-induced degradation.

### Electric and thermoelectric measurement on SAMs

A series of alkyl thiol-based self-assembled monolayers (SAMs) with varying chain lengths were fabricated, and their electronic transport properties were measured using a graphene-coated probe.

**Fig. 5:**
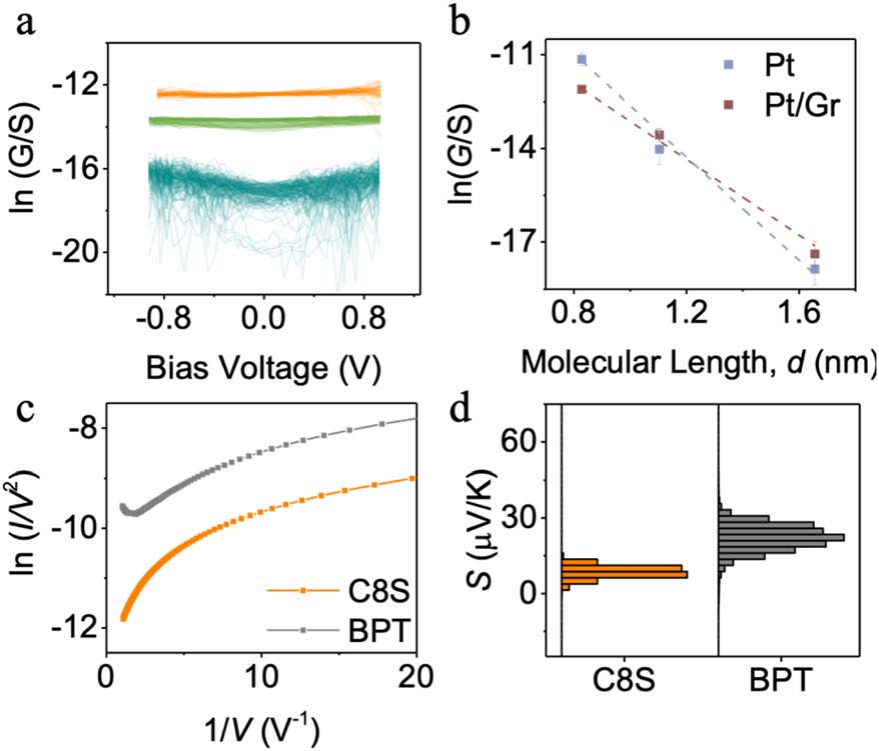
(a) Plot of log (*G*/*S*) *vs.* bias voltage for hexanethiol (C6S, orange), octanethiol (C8S, green) and dodecanethiol (C12S, Cyan) based SAM, measured by graphene coated probe. (b) Conductance decay over alkyl unit for using a Pt probe and a graphene coated probe. (c) Fowler–Nordheim plots and (d) distribution of *S*_junction_ values of C8S and BPT SAMs.

Fig. 5(a) shows the log (*G*) *vs.* bias voltage plots for alkyl thiol SAMs on Au^TS^ with 6, 8, and 12 CH_2_ units. As these chains consist of aliphatic carbon with a large HOMO–LUMO gap (∼7–9 eV),^46^ coherent tunnelling dominates the electron transport. Consequently, the molecular energy levels remain largely unaffected by changes in molecular length. The conductance *G* follows the relation:

1 ln(*G*) = ln(*G*_0_) − *βd*

where *G* is the measured conductance, *G*_0_ is the contact tunnelling conductance, *d* is the molecular length, and *β* is the decay factor associated constant associated with the tunnelling barrier.

Fig. 5(b) displays the plot of molecular length, *d*, *vs.* ln(*G*/*S*) for the SAMs, using both Pt and graphene-coated probes. The linearity confirms that tunnelling is the dominant transport mechanism. The decay constant, *β*, was higher for the Pt probe (8.4 nm^−1^) than for the graphene-coated probe (6.5 nm^−1^), consistent with earlier studies on alkyl-based junctions using graphene and metal electrodes systems.^30,47–50^ The contact conductance *G*_0_ for the Pt probe (∼10^−5^ S) was about an order of magnitude greater than that for the graphene-coated probe (∼8 × 10^−7^ S), as expected due to reduced interfacial coupling at the graphene/SAM interface.

It is important to clarify the physical origin of the observed changes in both the tunnelling decay constant *β* and the contact conductance *G*_0_ upon introducing the graphene interlayer. In the Au/SAM/Pt system, the electron transmission probability (*T*) is dominated by *T*_Au/SAM/Pt_. In contrast, for the Au/SAM/Gr/Pt systems, the transmission is dominated by *T*_Au/SAM/Gr_ × *T*_Gr/Pt_, where *T*_Gr/Pt_ accounts for the additional van der Waals gap between the physically transferred graphene and the Pt probe. This gap introduces an extra scattering barrier that is independent of SAM thickness, thereby reducing the overall contact conductance without affecting the length-dependent decay behaviour. The reduction in *β* from 8.4 nm^−1^ (Pt) to 6.5 nm^−1^ (graphene-coated) originates from improved electronic coupling at the SAM/probe interface. Compared to the σ–d coupling between the alkyl terminus and Pt, the σ–π coupling between the terminal CH_3_ group and graphene's π-system more effectively lowers the tunnelling barrier height. Thus, while the graphene layer enhances orbital overlap and reduces the barrier height (lower *β*), the van der Waals gap between graphene and Pt increases the effective barrier width, leading to a decrease in *G*_0_.

In addition to alkyl thiols, biphenyl thiol (BPT) SAM – featuring a conjugated backbone – were studied using the graphene-coated probe and compared with non-conjugated C8SH SAM of similar length. The conductance of BPT SAM was ∼30× higher than that of C8SH, due to π-conjugation and delocalised electrons in the phenyl rings that facilitate electron transport. Fig. 5(c) shows the Fowler–Nordheim (FN) plots for both SAMs. For BPT, a minimum in the FN plot at *I*/*V*^2^ = 1.58 V^−1^ was observed (corresponding to *V* = 0.63 V) indicates the transition voltage (*V*_T_), marking the shift from direct tunnelling to FN tunnelling. *V*_T_ provides an estimate of the energetic offset between the molecular orbital and the electrode's Fermi level, and the observed value aligns with those reported for oligo-phenyl systems in symmetric metal junctions.^51^ No such transition was observed for C8S, as its large HOMO–LUMO gap places the frontier orbitals far from the Fermi level.

While the FN plot estimates the energy difference between the molecular orbitals and the electrode Fermi level, it does not reveal whether the Fermi level lies closer to the HOMO or LUMO. Thermoelectric measurements can provide this information.^52–56^ These were performed on BPT and C8S SAMs using the graphene-coated probe, following the procedure described in the Experimental Section.

Before performing SAM measurements, the thermovoltage between the graphene-coated probe and the Au substrate at different temperatures was measured. The Seebeck coefficient between the graphene-coated probe and the Au substrate was calculated using the equation:

2 *S*_contact_ = −*V*_Therm_/Δ*T*

where *V*_Therm_ is the measured thermovoltage and Δ*T* denotes the temperature difference between the substrate and the probe (Fig. S7). The measured *S*_contact_ was −4 ± 0.8 µV K^−1^ (this value was 1.2 ± 0.2 µV K^−1^ for the Pt probe), indicating that the work function of the graphene probe was lower than that of gold.

For the SAM measurements, the Seebeck coefficient of the molecular junction was calculated using the equation:

3 *S*_junction_ = *S*_contact_ − *V*_Therm_/Δ*T*

Fig. 5(d) presents the distribution of *S*_junction_ values for BPT and C8S SAMs, while Fig. S8 shows the corresponding *V*_Therm_*vs.* Δ*T* plots. In both cases, a linear relationship was observed, with negative *V*_Therm_ and positive Δ*T*, indicating positive *S*_junction_. Since a positive value implies that the Fermi level is closer to the HOMO resonance. This is expected because the lone pair of thiols is co-planar with the π-system of the conjugated backbone. This planar configuration energetically positions the electrode Fermi level proximal down toward molecular HOMO. The calculated *S*_junction_ for BPT was 21 ± 5 µV K^−1^, significantly higher than that of C8S (8 ± 2 µV K^−1^). This agrees with the FN analysis: for BPT, the Fermi level lies ∼0.63 eV from the HOMO, while for C8S it is > 1 eV away, resulting in a steeper transmission slope and a higher Seebeck coefficient.

4 *S*_junction_ ∝ −*∂*_*E*_(In *T*(*E*))|_*E*=*E*_F__

Notably, despite the Au/SAM/graphene/Pt configuration used here, the measured Seebeck coefficients are almost identical to our previously measured Au/SAM/Pt junction with same setup, which give *S*_C8S_ = 7.4 ± 3.1 µV K^−1^ and *S*_BPT_ = 23.3 ± 7.3 µV K^−1^.^57^ This is because the interfacial coupling between Au/thiol is significantly stronger than graphene/SAM, which dominantly controlling the electrode Fermi alignment, while graphene layer only behave as interfacial layer but will not influence the molecular orbital alignment status, thus the Seebeck coefficient remain constant with and without graphene insertion.

## Conclusions

This study demonstrates the successful fabrication of graphene-coated conductive AFM probes using a scalable batch process. Compared to uncoated probes, the graphene-coated variants show superior wear resistance, improved data quality, and extended operational lifetime. The graphene layer effectively reduces friction and high-current-induced degradation, enhancing probe reliability.

Electrical and thermoelectric measurements on self-assembled monolayers reveal key insights into molecular transport properties. The graphene-coated probes deliver consistent, reproducible results, confirming stable measurement performance. Tunnelling-dominated transport observed in alkyl thiol-based SAMs aligns with previous findings, validating the coating's ability to preserve molecular energy levels and support coherent tunnelling. In biphenyl thiol (BPT) SAM, the presence of conjugated backbones enhances electrical conductivity. Comparison with C8S SAM confirms that the graphene-coated probes enable higher conductance in molecules with delocalised electrons.

Thermoelectric measurements highlight the energetic alignment between the electrode's Fermi level and molecular frontier orbitals. Positive Seebeck coefficients for BPT and C8S SAMs suggest proximity of the Fermi level to the HOMO resonance, resulting in steeper transmission near the Fermi level and enhanced thermoelectric performance. Long-term stability of the coated probes further supports their suitability for extended thermoelectric studies.

From a commercial standpoint, the cost-effectiveness of the proposed fabrication route is noteworthy. While the Langmuir–Blodgett (L–B) trough is not commonly available in typical laboratory settings, our approach supports a collaborative pathway where academic institutions equipped with L–B systems partner with AFM probe manufacturers for large-scale, reproducible graphene coating. This pipeline enables high-throughput production of probes with excellent uniformity at low incremental cost. Since the amount of graphene required per tip is minimal, the material cost for coating is negligible relative to standard AFM probe pricing. This scalable and economical coating strategy facilitates broader accessibility and supports future applications in molecular electronics without imposing substantial equipment burdens on end-users.

Overall, graphene-coated AFM probes offer notable advantages in durability, measurement fidelity, and molecular junction characterisation. This approach advances understanding of charge transport and thermoelectric behaviour in SAMs, providing a robust platform for future research in molecular electronics and nanoscale devices.

## Author contributions

BR, XW and OK conceived the idea to transfer graphene from a floating LB film to an AFM probe. XW led the experimental work with support from BP-J and AL for Langmuir–Blodgett deposition and probe characterisation, respectively. NA performed SEM characterisation. XW, BR and OK provided interpretation of the experimental data and theoretical analysis. XW drafted the manuscript, BR revised it and all authors contributed to the final version.

## Conflicts of interest

There are no conflicts to declare.

## Supplementary Material

NA-OLF-D5NA00924C-s001

## Data Availability

Data sets for this article, including all raw SPM data for Fig. 2–5, are available at Science Data Bank at [https://doi.org/10.57760/sciencedb.28293]. Supplementary information (SI) is available. See DOI: https://doi.org/10.1039/d5na00924c.
