# Supplementary material for: A graphene-coated AFM probe for durable and reproducible nanoscale electronic measurements
Source: Nanoscale Adv. 2026 Mar 4;8(8):2558–65. doi: 10.1039/d5na00924c (PMC12974596; doi:10.1039/d5na00924c)
Supplement: NA-008-D5NA00924C-s001 [file NA-008-D5NA00924C-s001.pdf]

Supplementary Information (SI) for Nanoscale Advances.
This journal is © The Royal Society of Chemistry 2026

Electronic Supporting Information (ESI)

A Graphene-Coated AFM Probe for Durable and Reproducible Nanoscale Electronic Measurements

Xintai Wang, [a,b] Angelo Lamanita, [a] Becky Penhale-Jones, [a] Nema Abdelazim, [c] Oleg. V. Kolosov, [a] and Benjamin. J Robinson [a*]

[a.] Physics Department, Lancaster University, Lancaster, LA1 4YB, United Kingdom. E-mail: b.j.robinson@lancaster.ac.uk
[b.] Zhejiang MaShang GM2D Research Institute, 366 Shanhai Street, Cangnan, Wenzhou, Zhejiang China and School of Information Science and Technology, Dalian Maritime University, Dalian, China.
[c.] School of Electronics and Computer Science, University of Southampton, Southampton SO17 1BJ, United Kingdom.

Table of Contents

Fig. S1 – Further details of graphene transfer process

Fig. S2 – AFM characterisation of graphene film

Fig. S3 – SEM images of coated and uncoated probes

Fig. S4 – SEM images of coated and uncoated probes after AFM scanning

Fig. S5 – Young's modulus of a coated and uncoated probe

Fig. S6 – Current map of BPT SAMs using coated and uncoated probes

Fig. S7 – Thermovoltage distribution of a graphene coated probe on Au^{TS}

Fig. S8 – Thermovoltage distribution of BPT and C8S SAMs measured by graphene coated probe

Fig. S9 – Further details of probe 'de-wearing'

Fig. S10 – Lifetime measurement of coated and non-coated probes

Table. S1 – Lifetime measurement of coated and non-coated probes

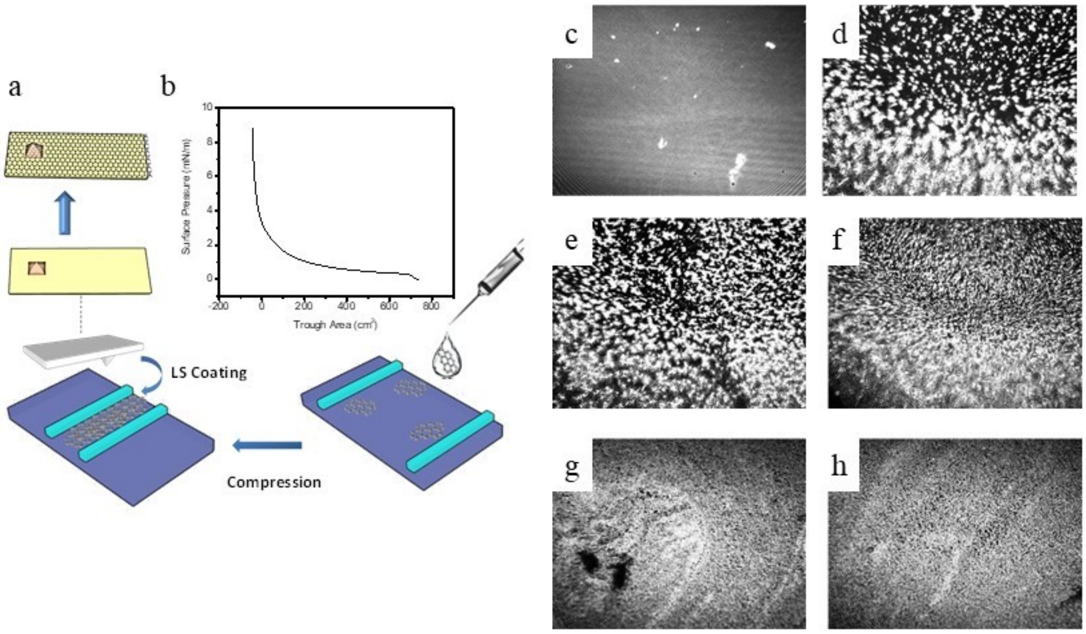

Fig. S1 (a) An illustration of a conductive-AFM probe coated by graphene layer, (b) and the

isotherm spectra for LB film formation from graphene nano-flakes. (c-h) KSV photo of LB trough surface during compression while surface pressure is 0, 1, 2, 4, 6 and 8 N/m.

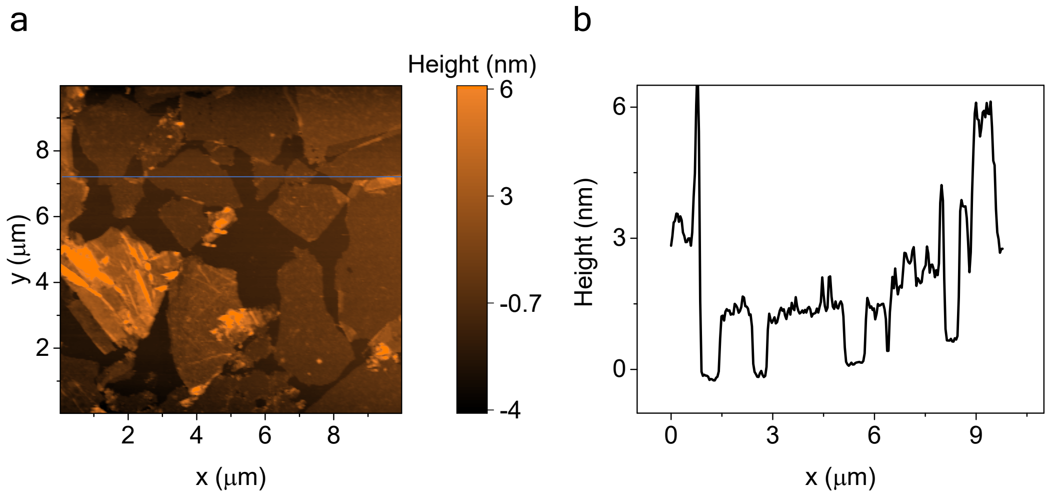

Fig. S2 (left) AFM image of graphene film prepared by LB method on Si (right) corresponding height profile of graphene flakes.

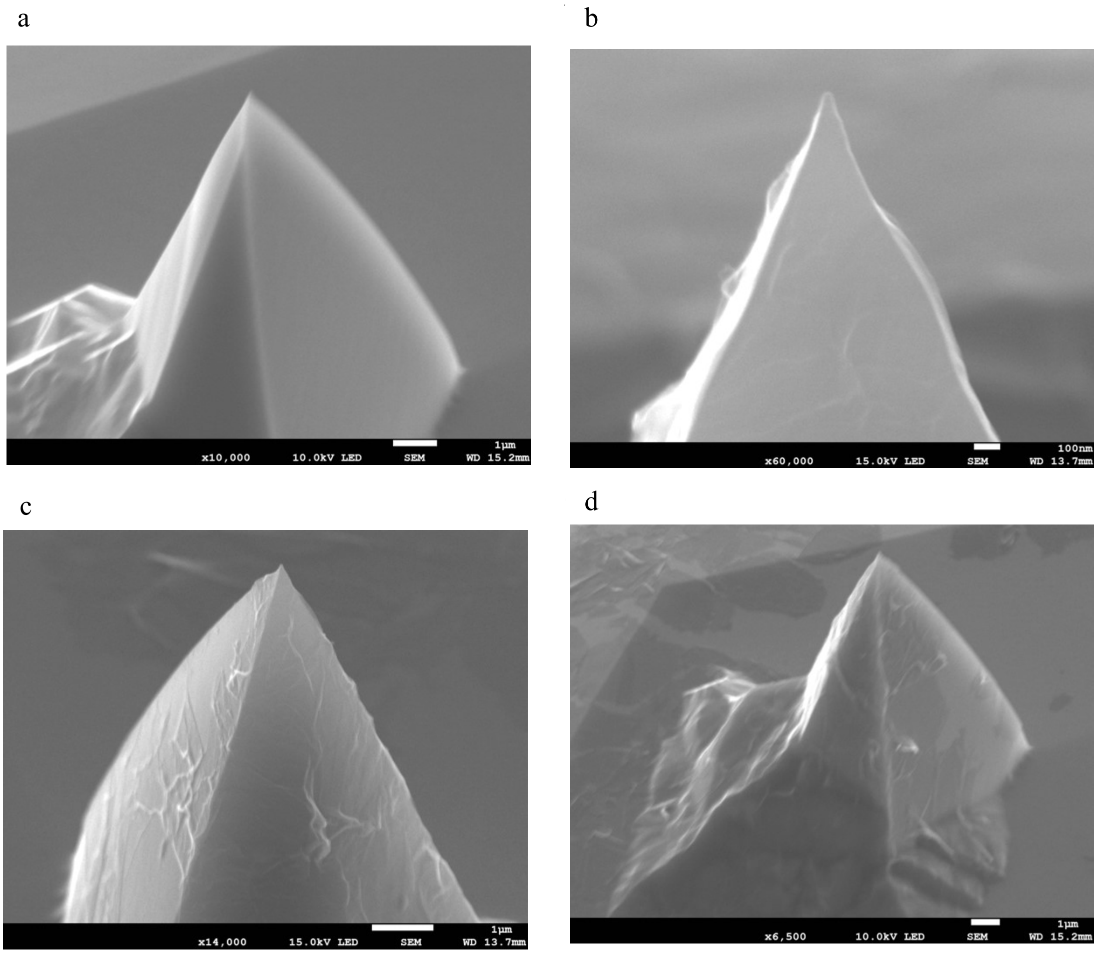

Fig. S3 (a) SEM image of a new pt coated probe. (b, c, d) SEM image of graphene coated probes

prepared from different batches.

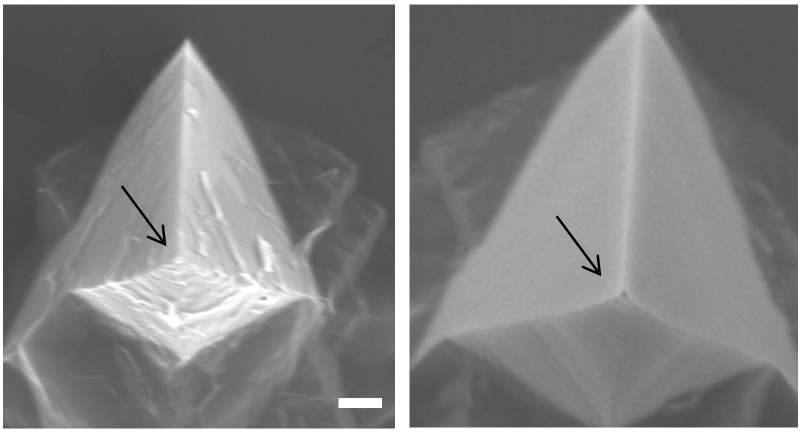

Fig. S4 (left) SEM image of a graphene coated probe after 5 cycles of image scan. (right) SEM image of a pt probe without graphene coating after 5 cycles of image scan, black arrow indicate the de-wearing part at apex.

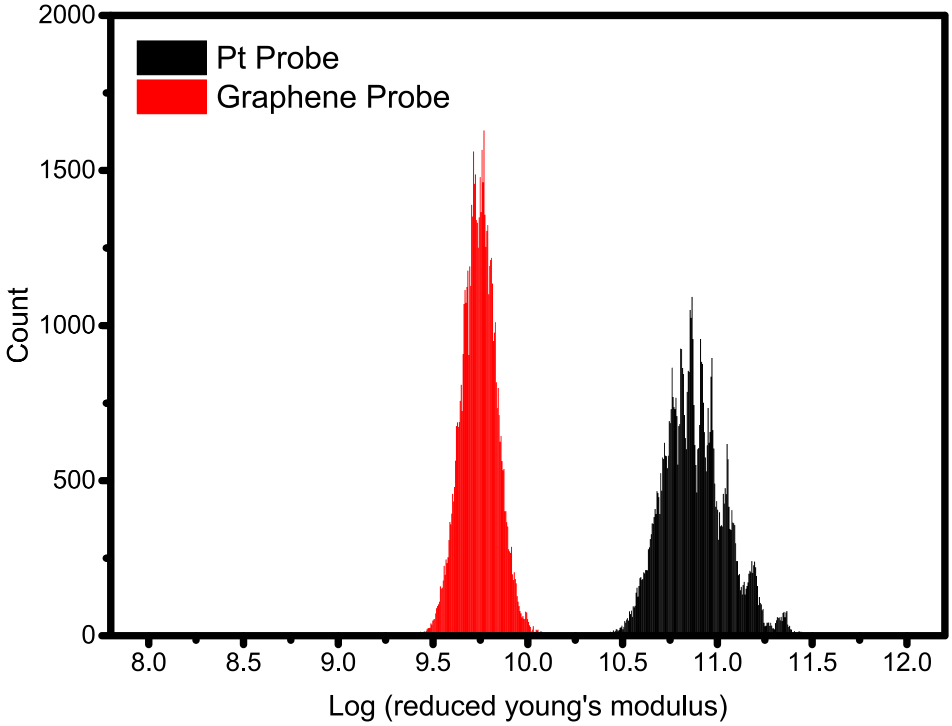

Fig. S5 Young's modulus of a Pt probe and a graphene coated probe contacting an Au^{TS} substrate, the significantly lower young's modulus for graphene coated probe means graphene was acting as a soft top contact to SAMs underneath.

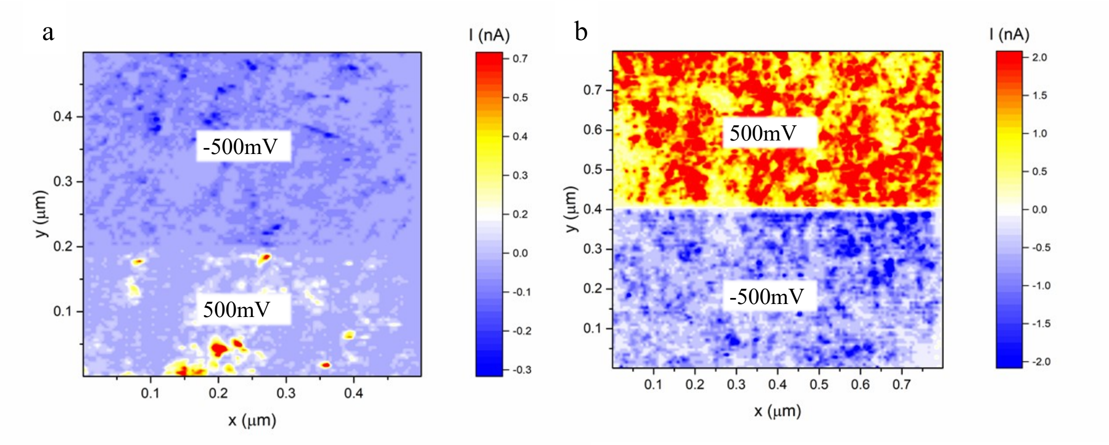

Fig. S6 Current map of BPT SAMs using a normal Pt probe (a) and a graphene coated probe (b), bias voltage = 500 mV and -500mV.

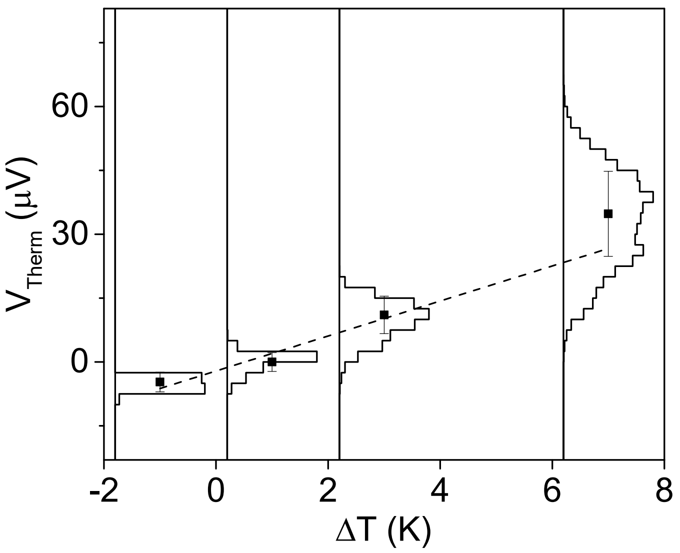

Fig. S7 Thermovoltage distribution of graphene coated probe on Au^{TS} and ΔT, positive relationship between thermovoltage and ΔT indicate negative Seebeck coefficient.

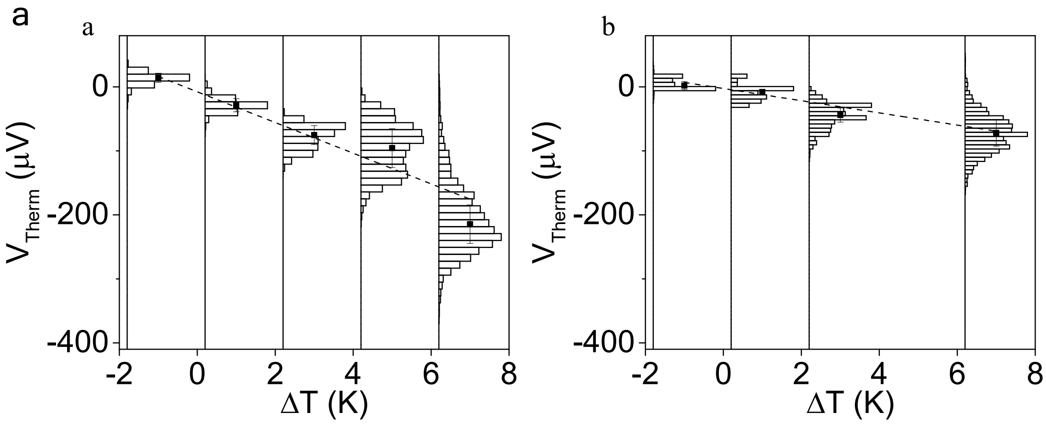

Fig. S8 Plot of thermovoltage distribution of BPT (a) and C8S (b) SAMs measured by graphene coated probe vs. ΔT, negative relationship between thermo-voltage and ΔT indicate positive Seebeck coefficient.

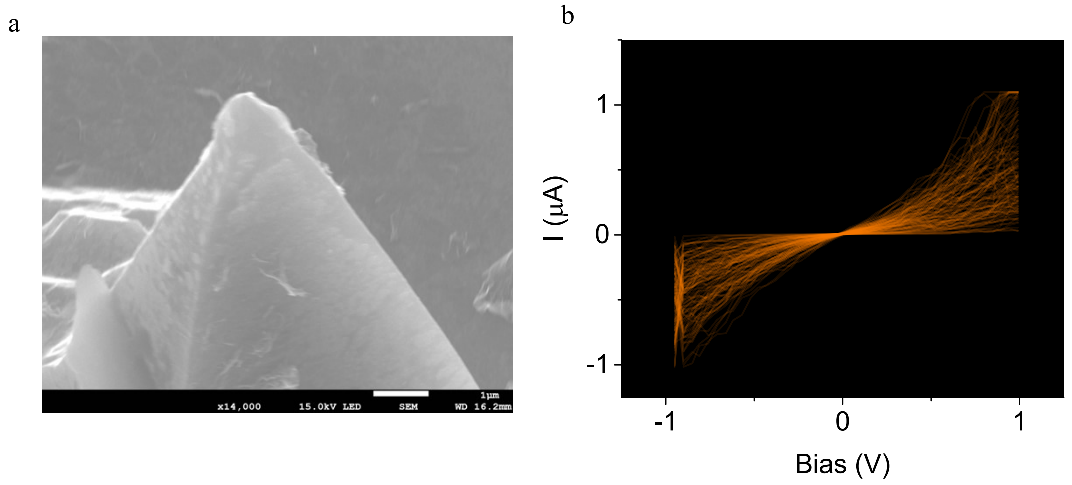

Fig. S9 IV curve statistics for BPT SAMs (right), measured by a graphene coated de-wearing probe (left).

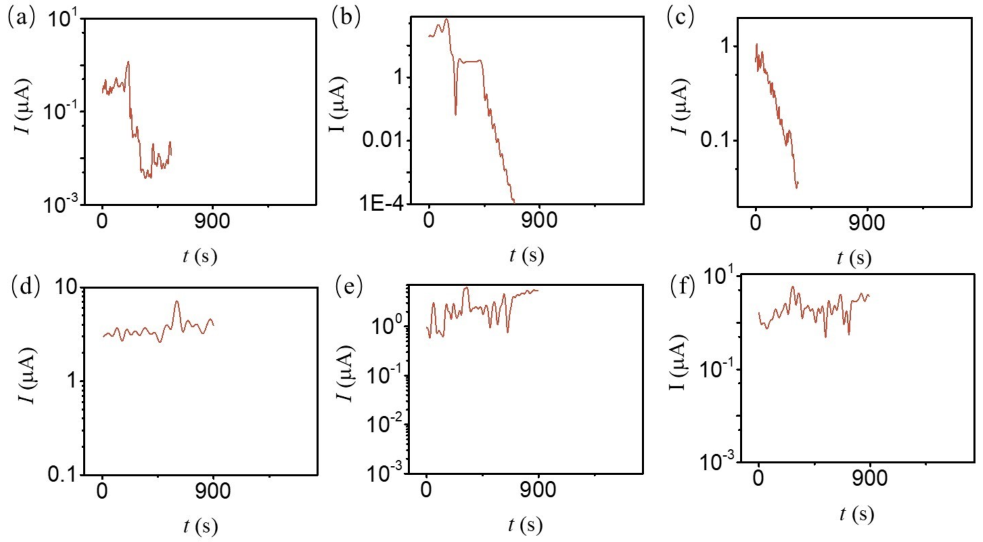

Fig. S10. Lifetime measurements of conductive probes with and without graphene coating. (a–c) Current stability tests for three individual probes without graphene coating. (d–f) Current stability tests for three individual probes with graphene coating. All measurements were performed under conditions identical to those in Fig. 4.

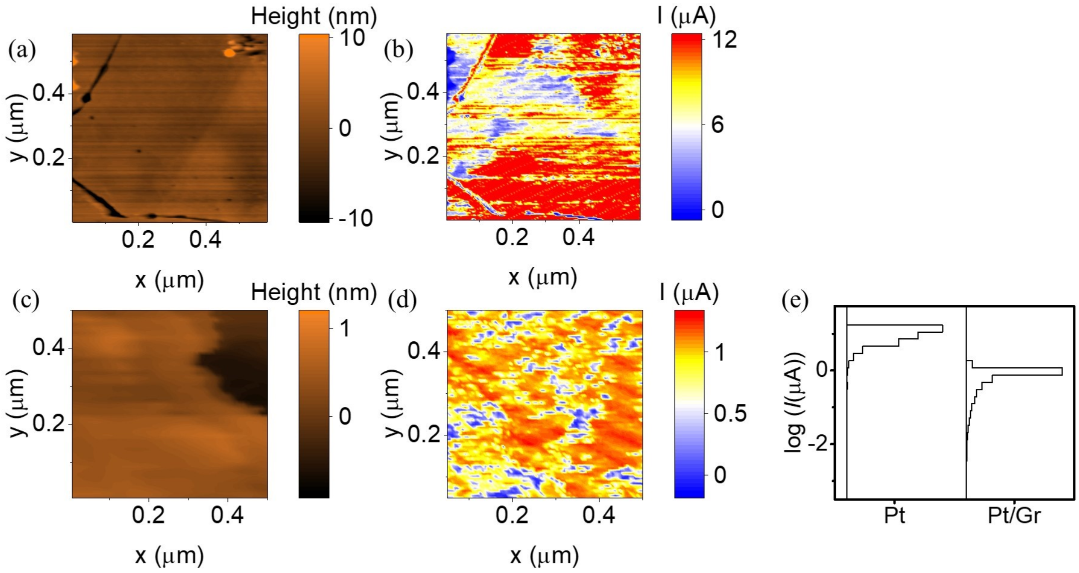

Fig. S11. AFM topography and current map of template striped gold scanned using Pt probe without (a, b) and with (c,d) graphene coating, and the current distribution of current map (e).

Table S1. Life-time of Pt AFM probe with and without graphene coating

	Probe life-time (s)*
Pt - 1	210
Pt - 2	280
Pt - 3	240
Pt - 4	190
Gr/Pt - 1	>900
Gr/Pt - 2	>900
Gr/Pt - 3	>900
Gr/Pt - 4	>900

* Probe life-time indicate the time while current decreased for 10 times (t at $I_{initial}/I = 10$)
